# Trait networks: Assessing marine community resilience and extinction recovery

**DOI:** 10.1016/j.isci.2024.110962

**Published:** 2024-09-16

**Authors:** Charlotte G. Clay, Alexander M. Dunhill, James D. Reimer, Maria Beger

**Affiliations:** 1 School of Biology, Faculty of Biological Sciences, University of Leeds, Leeds LS2 9JT, UK; 2 School of Earth and Environment, Faculty of Environment, University of Leeds, Leeds LS2 9JT, UK; 3 Molecular Invertebrate Systematics and Ecology Laboratory, Graduate School of Engineering and Science, University of the Ryukyus, 1 Senbaru, Nishihara, Okinawa 903-0213, Japan; 4 Tropical Biosphere Research Center, University of the Ryukyus, 1 Senbaru, Nishihara, Okinawa 903-0213, Japan; 5 Centre for Biodiversity and Conservation Science, School of Biological Sciences, University of Queensland, Brisbane, QLD 4072, Australia

**Keywords:** Paleontology, Ecology, Paleobiology

## Abstract

Extensive global habitat degradation and the climate crisis are tipping the biosphere toward a “sixth” mass extinction and marine communities will not be spared from this catastrophic loss of biodiversity. The resilience of marine communities following large-scale disturbances or extinction events is mediated by the life-history traits of species and their interplay within communities. The presence and abundance of traits in communities provide proxies of function, but whether the breakdown of their associations with species loss can delineate functional loss remains unclear. Here, we propose that relationships between traits within trait networks provide unique perspectives on the importance of specific traits, trait combinations, and their role in supporting the stability of communities, while exploring the vulnerability of both past deep time and present-day marine communities.

## Graphical abstract

**Figure:**
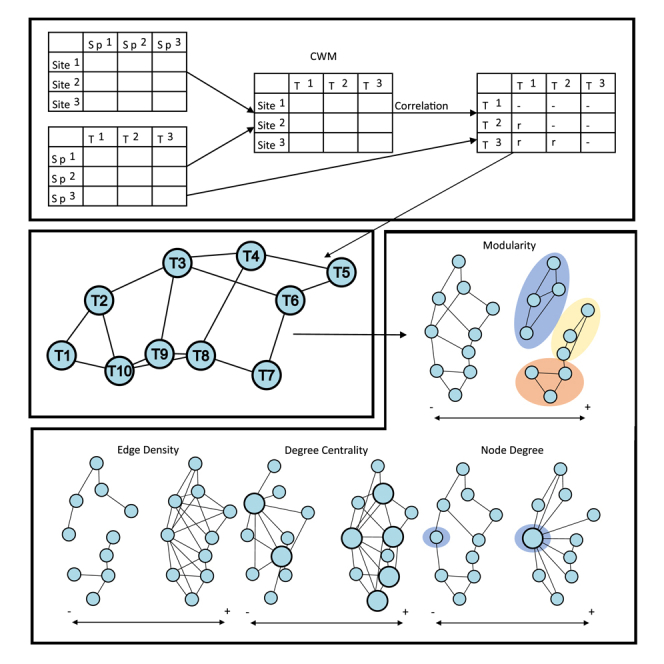

## Introduction

The diversity of marine life is constantly changing, with present-day ecosystems being shaped by past and ongoing disturbance and extinction events. Evaluating how the structure of marine communities and ecosystems will change in response to climatic shifts are crucially important for understanding ecological responses to climate change in the future.^1^^,^^2^^,^^3^^,^^4^ As environmental change drives marine species toward regional or global extinction, evaluating ecosystem responses to past extinction events may help predict possible ecosystem futures, bridging the gap between community ecology and paleobiology.^5^ Many past marine extinctions were driven by environmental stressors, such as rapid warming, anoxia, or acidification. Many of these extinctions were of a greater magnitude than the current event, but stressor type and levels were possibly equivalent to potential future climate change conditions. Thus, such events can provide potential insights into how community assemblies may change in the future.^6^^,^^7^^,^^8^ Although rates of change may differ between past and present environmental changes, the resulting ecological processes of community reorganization, resilience, and extinction events can still provide the closest analogies to how modern communities may change in the future, as the levels of climatic changes being faced are unprecedented in both written history and archaeological records.^9^

Ecological resilience is often defined as the ability of an ecosystem to retain its structure and function following a disturbance event.^10^ High species richness underscores the ability of a community to retain function under long-term perturbations, and therefore the resilience of that community.^11^ Loss of species in a community due to regional or global extinctions can, therefore, reduce the resilience of a community. In cases where functionally important species are lost, or mass extinction events have occurred, the effect on community resilience will be of greater magnitude.^12^ Species traits can be linked to functioning,^13^ therefore, trait-based analysis can identify functionally important species and quantify how community assemblies have been or will be affected by the loss of such species.

Traits can improve a species’ adaptation potential to environmental change or its survival probability during a mass extinction event.^7^^,^^14^^,^^15^ Although reef collapses have been a common feature of Phanerozoic mass extinctions and their recovery is often slow, some specific scleractinian coral traits confer increased survival of extinction events.^14^ For example, traits such as being solitary, bleaching resistant, and having a cosmopolitan distribution improve coral survival probabilities during an extinction event.^14^ Communities made up of species with resilient trait combinations increase the likelihood of whole community resilience. High biodiversity reinforces the resilience of communities to disturbances,^12^ along with functional redundancy^16^ and high response diversity^17^ in communities. Functional redundancy can act as an “insurance policy”, as when several species inhabit a functional niche, the loss of one species will not lead to the loss of the function it provided within the community.^15^^,^^16^^,^^18^ However, even highly diverse communities are often more functionally vulnerable than has been thought due to niches in highly diverse communities being relatively narrow.^19^ It is also important to consider response diversity, if multiple species occupy the same niche space but also respond in the same way to environmental changes then functional redundancy may still be low.^17^

Species and trait losses during ongoing environmental changes may have different consequences on community structure and functioning than analogous species and trait losses on paleontological time scales. Marine species extinctions have been documented throughout the fossil record with extinction rates ranging from a very low “background level” to major mass extinctions where over 75% of marine species disappeared from the fossil record.^20^^,^^21^^,^^22^ Extinction rates vary greatly both temporally and phylogenetically, and current background extinction rates are estimated to be much higher than those in the recent fossil record.^21^ The magnitude and rates of species richness and abundance losses will increase as the climate crisis intensifies, further exacerbating ongoing and unprecedented global ecological transformations.^21^ When using paleontological data to investigate the restructuring of communities due to environmental changes, it is important to consider how time frames differ between deep time and modern community change for accurate comparisons to present-day data.^23^ Focusing on magnitudes or processes of change, rather than rates of change, provides a promising way forward in paleobiological research^8^ by making paleobiological and modern communities more comparable.

Species co-occurrence networks can reveal the complicated structure of communities and the interactions between species within those ecosystems.^24^^,^^25^^,^^26^ The diversity of species associations is important for community resilience and stability.^27^ Species co-occurrence networks can reveal trophic and non-trophic ecological interactions.^24^^,^^25^^,^^28^ However, their main strength is their ability to quantify relative changes in functioning, allowing the visualization of the turnover of positive and negative associations between species. This strength is especially evident when co-occurrence networks are combined with trait data.^26^^,^^29^

Trait-based techniques can support characterizing and predicting the vulnerability of marine species to climate change,^30^ anthropogenic threats,^31^ loss of functional diversity, and community turnover along environmental gradients.^32^^,^^33^^,^^34^^,^^35^ Moreover, they present a promising framework for predicting future functional changes.^36^ The integration of trait-based techniques and network theory provides a promising route to understanding changes in trait combinations and correlations^37^ following environmental changes. The combination of trait-based analyses and co-occurrence networks to create trait co-occurrence networks reveals trait-trait correlations,^37^ and when applied to marine community ecology as we suggest in the further section, can allow for a comprehensive picture of community resilience to environmental changes, encompassing species and trait turnovers and allowing the quantification of functional changes.

Although the combination of traits and co-occurrence networks is not new to marine science (e.g., Siwicka et al., 2020^38^), direct trait co-occurrence networks have rarely been constructed for marine communities (but see in the study by Gladstone-Gallagher et al., 2023^39^), despite important extinction and disturbance-driven changes occurring in these communities, both in the past and the present. Thus, we propose using trait co-occurrence networks, hereafter referred to as trait networks, to assess community resilience to disturbances in both modern and deep-time contexts, while being aware of the discrepancies in temporal scales between data from contemporary ecological studies and that which is housed in the fossil record. By examining broad changes within trait co-occurrence networks, comparisons between community and trait assemblage alterations can be made, and we provide two separate examples of the application of trait networks, one each to modern and fossil communities (Boxes 1 and 2). Trait-based network theory allows for the exploration of deep-time community changes and vulnerabilities without the confusion of modern-day anthropogenic disturbances, allowing the isolation of climate change effects.^8^ Quantifying the magnitude and processes associated with these climatic-driven community changes, such as the loss of function within communities, can facilitate more accurate predictions of the magnitude of future changes.^8^

## Constructing trait networks

**Figure 1:**
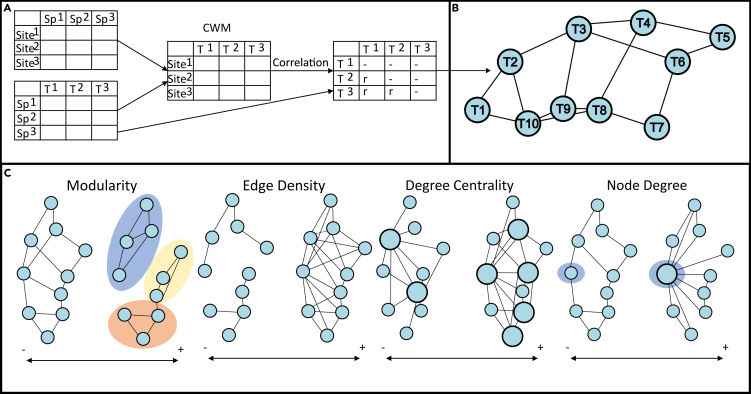
Proposed approach to building trait networks for marine communities (A) Combination of a site by species (presence/absence or abundance/biomass) matrix and a species by trait matrix to create a site by trait matrix of community weighted means (CWM), followed by the establishment of a trait-trait adjacency matrix. Where site/transect information is unavailable, correlation metrics can be applied directly to the species by trait matrix. (B) Visualization of the correlation matrix as an undirected network. (C) Suggested network metrics to explore the resilience of the community (modularity: the measurement of how many modules, or distinct sub-networks, are formed within a network, where a module is defined by traits that have more connections with each other than with other traits within the network, edge density: indicates how well-connected the overall network is, and degree centrality: the number of highly influential traits within the network) and identify important traits (node degree: the importance of the node within the network).

Trait networks visualize trait-trait correlation matrices as an undirected network, where nodes are traits and edges are trait-trait relationships.^37^ Using trait networks to explore the links between morphological, life-history, and functional traits across tissues, organisms, and communities is a rapidly advancing approach within plant science,^37^^,^^38^^,^^40^^,^^41^^,^^42^^,^^43^^,^^44^ but is also easily transferrable to marine questions. The construction of a trait network involves several steps. A presence/absence trait matrix is required for all species across multiple sites, which is then combined with a site-by-species matrix. If the site-by-species matrix contains abundance data, community-weighted means (CWM) can be calculated as this allows numerical values to be assigned to each trait and a more accurate perspective of trait prevalence within a community.^45^ Using CWMs to create trait-trait correlation matrices may not be a practical approach for all fossil data, due to the variation in spatial scales of fossil occurrence data and the lack of study sites or transects, along with uncertainty, derived from preservation bias, around how abundances from fossil assemblages reflect the actual abundances of organisms within a community. A trait-trait relationship matrix is created by the combination of the trait matrix and site by species presence/absence or CWM matrix often using correlation or probabilistic methods.^37^^,^^40^^,^^43^^,^^44^ Generally, a threshold is set (e.g., r = 0.2) where correlations above this threshold are assigned as 1 and those below are assigned as 0, therefore indicating the presence and absence of significant trait-trait relationships.^37^ Therefore, trait co-occurrences do not always equal trait-trait relationships or correlations, which brings in the potential to overlook some relationships not deemed as significant, this is particularly true when using paleontological data where abundance data are unreliable, and certain species/traits are likely missing. The resulting trait-trait matrix can then be visualized as a network of correlations and standard network metrics can be calculated^37^^,^^40^ (Figure 1). This methodology identifies traits that co-occur across sites and thus explores ecological shifts in space and time that are more broadly relevant to function and resilience than shifts in species composition.

Network metrics reveal links between traits, allow for comparisons between different networks, identification of important, well-connected traits and have the potential to reveal how networks change following the loss of an important trait.^37^ There are many different network metrics, and we highlight those that we feel are most relevant to the resilience and recovery capacity of a community from a disturbance, based on our assumptions that trait and species co-occurrence networks can be interpreted similarly. For assessing the structure of overall trait networks, modularity, edge density, and degree centrality are important, whereas node degree identifies nodes (i.e., traits) that are central to the network^40^^,^^43^ and may hold a keystone role. Network modularity is the measurement of how many modules, or distinct sub-networks, are formed within a network, where a module is defined by traits that have more connections with each other than with other traits within the network.^40^^,^^46^ In species co-occurrence networks, high levels of modularity buffer against the cascading effects of a disturbance,^46^^,^^47^ as when not all nodes within the network are connected, there is a higher chance of adaptation or evolution in response to environmental change.^40^ Modularity in species co-occurrence networks has also been used to identify groups of species with similar responses to environmental change,^48^ and high levels of modularity have been linked to increased redundancy in communities.^46^ Therefore, when looking at connections between community-level traits increased modularity likely translates to increased functional diversity and/or response capacity, depending on the traits used, and therefore increased resilience to change.^12^^,^^16^^,^^17^^,^^39^ Edge density is the proportion of trait-trait relationships identified as significant out of all logically possible connections within the network.^49^^,^^50^ Therefore, edge density indicates how well-connected the overall network is. Highly connected trait networks may be favored in marginal conditions,^40^ where trait diversity is reduced and abundant generalist life strategies render trait matching more likely,^35^ but responses can be variable. For example, when looking at macroinvertebrate response traits, increased network complexity reduced the variability of response to nutrient stress.^39^ The response of less complex networks was far more variable, as some simple networks were made up of a few very resilient connections and others were made up of a few vulnerable connections,^39^ highlighting the importance of understanding community makeup and dynamics when assessing community resilience. Degree centrality measures the number of highly influential traits within the network.^43^ Networks with a low degree of centrality, i.e., where only a few key traits influence the network structure, are linked to high sensitivity to disturbances, as the loss of one or two key traits could break down the network.^43^^,^^46^ Node degree relates to the number of edges connecting one node to another, where traits with a high node degree can be thought of as keystone traits.^37^ Identifying keystone traits allow the resilience of the network to be investigated by looking at the vulnerability of the keystone traits. The vulnerability of networks with only a few hub traits will depend on the traits chosen and the vulnerability of the species they relate to. Networks with only a few hub traits are often thought of as vulnerable to disturbance,^40^^,^^43^ however, as with the connectivity example aforementioned, if the hub traits are resilient traits the network may not be vulnerable. Therefore, we propose networks with high modularity, edge density, and degree centrality indicate comparatively higher resilience within a community than networks with lower network metrics. However, knowledge of the dynamics of the study community is also required to assess the resilience of a community properly.

Once a trait network is constructed and its metrics are calculated, it is important to assess the significance of these metrics. Several methods to evaluate metrics exist, and the choice will depend on the ecological question at hand. Here, we outline two approaches: random networks and bootstrapping.

To determine if trait co-occurrences significantly differ from random networks, we can create random networks and compare them to the observed network.^49^ For example, the Erdos-Renyi model^51^ can generate random networks with the same number of nodes and edges as the observed network but with a randomized structure.^52^ The observed network metrics can then be compared to the random network metrics using a permutation test.^53^

To test whether the metrics of two networks, such as pre-and post-disturbance networks, are significantly different, bootstrapping can be used. This involves creating 1,000 bootstrapped trait datasets and generating networks from each.^40^ By bootstrapping each dataset, creating 1,000 networks, and calculating metrics for each time period or area of interest, the two groups of networks can be compared. This comparison can apply 95% confidence intervals^40^ or independent sample t-tests^44^ to assess if the network metrics differ significantly.

## Trait choice to evaluate community change

Trait choice has been a highly debated topic in trait-based ecology, especially when it comes to deciding which traits relate to functioning.^54^ The choice of traits selected for a particular research question depends largely on what that question is. Streit and Bellwood (2023)^54^ suggested a framework they call the “Taxonomy of Traits”, which split traits into Community Cluster Traits and Ecosystem Function Traits. If a research question aims to quantify diversity, trait combinations and what functions are available within a community, Community Cluster Traits would be useful (e.g., Nalley et al., 2024^55^), whereas if the research question is about specific functions and their impact on the ecosystem, Ecosystem Function Traits would be useful, i.e., traits that act as a direct proxy for function (e.g., Denis et al., 2024^56^).

Trait specification and the parameterization of trait levels differ by taxa. Whereas plant studies measure morphological trait variances both within and among species to create trait-trait relationship matrices,^43^ most studies in coral reef science can only estimate among-species trait variance. Within marine communities, it is typically impractical to measure how morphological traits vary due to the inherent difficulty in underwater fieldwork and accessibility to species, as well as the movement of species. The relationship between species traits and function is also more defined in plant science^40^^,^^43^^,^^44^ than in marine science due to the high feasibility of testing proposed relationships in plants. Further, it is beneficial to include behavioral and reproductive strategy traits when using animals in trait-based analyses^19^^,^^35^^,^^57^^,^^58^ (but see in the study by Schleuning et al., 2023^59^). In contrast, with paleontological data, measured morphological traits of fossils are likely to be the only available traits,^9^ although some traits can be inferred from modern analogs from the same genus or family or via functional morphological inference.^15^^,^^60^^,^^61^^,^^62^^,^^63^^,^^64^^,^^65^^,^^66^^,^^67^ As well as considering the relevance of chosen traits to the question at hand and the traits available, the number of traits chosen should also be considered in order to avoid including too few traits and producing artificial functional redundancy, or choosing too many traits and inadvertently including overlapping or irrelevant traits.^68^^,^^69^

The co-occurrence of a range of functional groups likely improves community functioning, where functional groups are defined as groups of species that share morphological and physiological traits.^70^ For example, in coral reef communities, the co-occurrence of many herbivorous fish functional groups increases coral cover and richness.^71^ Diverse marine communities support higher niche partitioning than communities of lower species richness,^13^^,^^72^ analogously, the diversity of traits will likely be positively correlated with community functioning. Furthermore, traits are not independent of each other^40^ but are often intercorrelated and determined by ecological factors^40^ within communities, with the presence/absence or abundance of some traits relying on others. Traits can be correlated due to life history strategies, for example, where multiple species share strategies such as reproductive output and offspring size based on variation in environmental conditions.^73^ For example, in reef fish community reductions, pelagic larval durations (PLD) are often correlated with increasing latitude,^35^ reflecting the fact that higher temperatures allow for shorter larval durations.^74^ The co-occurrence and intercorrelated nature of traits and trait combinations can be analyzed using trait networks and their metrics, which signifies how resilient the network is to disturbance or the loss of a trait or trait combinations. Furthermore, the number of different traits used may also affect the patterns seen, as many traits can be redundant to each other. Using large quantities of traits, such as some plant trait networks have,^44^ may lead to high redundancy and loss of the network “message”. Eklöf et al. (2013)^75^ suggested that for niche theory models of trophic interactions, the selection of three to five traits is optimal, and further analyses are needed to assess if similar constraints apply to trait networks. Plant trait networks often focus on functional traits and their relationship to ecosystem functioning,^40^^,^^43^^,^^44^ however, particularly for paleobiological data, only morphological trait data are available for coral reefs and thus the link to function is tenuous. This issue limits the questions that can be answered with trait networks, although morphological traits have been linked to increased extinction risks in some marine species (e.g., corals^9^), indicating a potential connection to resilience. When using categorical traits, the scale of trait categories should also be considered, as coarser or finer trait subdivisions will change the number of traits within the network and therefore their relationships. For example, finer trait subdivisions will appear to reduce functional redundancy, as more subdivisions mean less functional overlap and likely split degree centrality across more nodes.

## Application of trait networks to understand community responses to disturbance

Trait networks can characterize the robustness of functions across environmental gradients,^40^ assess the vulnerability of ecosystems to environmental change,^43^ and adaptations to changing environmental conditions.^44^ Trait networks reveal essential traits, trait-trait relationships, and ecosystem components (e.g., resource availability) required for the adaptation to changing environmental conditions.^40^^,^^43^^,^^44^ For example in plants, trait networks have revealed a trade-off between root and leaf traits in arid areas,^43^ indicating plants that favor leaf traits may have increased vulnerability to drought.

Unpicking the meaning behind trait-trait correlations can be complex and often network, community, and ecosystem dependant. Here, we provide examples of marine trait networks to explore ecosystem transformations from local extinctions in marine systems with the methodology discussed previously (Figure 1). First, we demonstrate how trait-trait relationships between tropical and temperate modern reef fish communities on the Pacific coast of Japan vary (Box 1). Similarly, our second example assesses paleobiological resilience with occurrence and trait data from Dunhill et al., (2022).^67^ This dataset contains shallow marine fossil species occurrence and their traits from across the Early Toarcian Extinction Event (ETEE) in the Cleveland Basin, UK (Early Jurassic, ∼183 Ma) (Box 2).

Box 1 **Trait networks for modern fish communities: Shifts in the trait networks of reef-associated fish along a tropical to warm temperate gradient**

Temperature naturally dictates the distribution of species,^79^ with the tropics generally harboring greater diversity and more functional redundancy. We assessed the changes in trait correlations between tropical and temperate reef-associated fish communities to evaluate if higher diversity equals higher resilience. To investigate variation in trait-trait relationships at sites along the tropical to warm temperate gradient in Japan, we recorded the biomass and five traits for 183 reef fish species that associate with coral communities. The traits chosen were maximum length, pelagic larval duration, trophic level, water column position, and reproductive mode, chosen to represent the morphological, behavioral, and biological functional niches of reef fish^35^ (Table 1).

We calculated CWM for each trait value for each site. We chose two of the environmental site clusters from Clay et al., (2024)^35^ (Tropical and Temperate) to represent extremes of the environmental gradient and calculated Pearson’s correlation matrices for each region. We then visualized the correlation matrices as trait networks. The trait database for each region was bootstrapped 1000 times and network metrics were calculated and compared for each of the 2000 networks produced (S1: Appendix).

**Figure 2:**
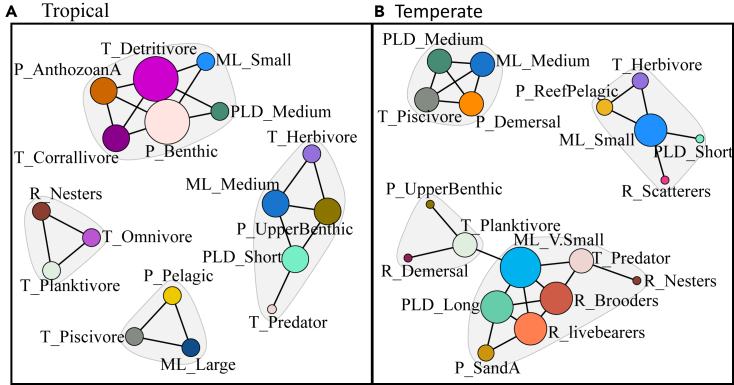
Reef fish trait networks at Tropical and Temperate sites along an environmental gradient in Japan (A) Tropical trait network. (B) Temperate trait network. Nodes = traits, Colors = individual traits and ellipses = modules. Node sizes scale with their standardized degree of centrality. Black lines indicate significant (*p < 0.05*) links between traits. Trait categories: ML = maximum length, PLD = pelagic larval duration, T = trophic level, P = water column position, R = reproductive mode.

There is a turnover in traits between the tropical and temperate networks, from specialist traits such as anthozoan-associated, corallivores, and nesters being important in the tropics to more generalist traits such as predators and scatterers in the temperate region (Figure 2). Degree centrality was significantly lower in the temperate region (Median = 0.57 (IQR = +/− 0.40)) than in the tropical region (Median = 0.64 (IQR = +/− 0.34)), indicating this region may be highly impacted by disturbances (U = 539896.5, *p* < 0.01), but further investigations into the resilience of the traits, connections and species present in the community are required to confirm this. Edge density and the number of modules were not significantly different between the two regions.

**Table 1:** The five traits used to investigate variation in trait-trait relationships along the Pacific coast of Japan and their ecological and functional relevance

Trait	Data Format	Ecological relevance of traits	Effect on ecosystem functioning
Maximum length	4 categories: very small (0–10 cm), small (11–50 cm), medium (51–100 cm), large (<101 cm)	Size relates to energy requirements of a species and its predator-prey relationships.^19^	Linked to regulation of food webs and nutrient cycling.^80^
Pelagic larval duration (PLD)	4 categories: short (0–40 days), medium (41–80 days), long (81–120 days), very long (<121 days)	Important early life history trait, relates to larval growth rates and ultimately individual survival.^81^	Relates to individual fitness, therefore to overall fish biomass and community composition. This will, in turn, affect ecosystem functioning.^68^
Trophic level	6 categories: detritivore, herbivore, planktivore, piscivore, predator, corallivore.	Different trophic levels impact ecosystem functioning at different levels.^19^	Varying trophic impacts on fish biomass, regulation of food webs and nutrient cycling.
Water column position	8 categories: benthic, anthozoan associated, demersal, pelagic, reef pelagic, sand associated, sub benthic, upper benthic,	Affects availability of prey and ecological niche.^19^	Differing substrates affect productivity and nutrient cycling in different ways.^82^
Reproductive method	5 categories: brooders, demersal, nesters, scatterers, live bearers	Important life history trait.^81^	Relates to individual survival and thus ecosystem functioning.^68^

The format used for each of the trait approaches is also shown. Modified from Clay et al. (2024).^35^

Box 2 **Trait networks for fossil communities shifts in the trait networks of marine communities during an extinction event**

**Figure 3:**
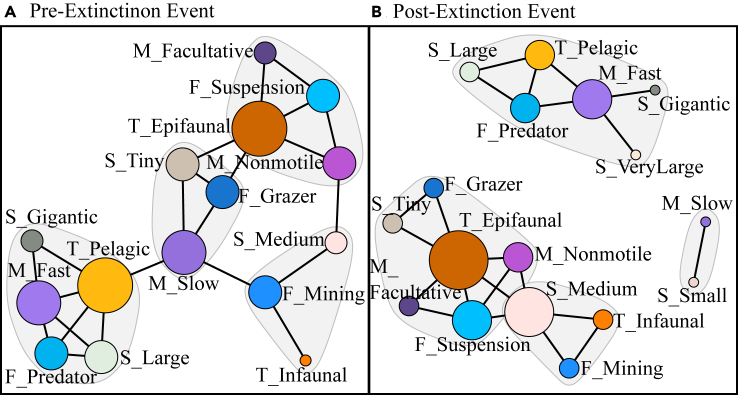
Shift in the trait of shallow marine communities during the early Toarican Extinction Event (A) *Pre-extinction* event trait network. (B) *Post-extinction* event trait network. Colors and ellipses indicate individual traits and modules respectively. Node sizes scale with their standardized degree of centrality. Black lines indicate significant (*p < 0.05*) links between traits.

Extinction events lead to the loss of species due to environmental stress and secondary extinction cascades, causing the restructuring of community and trait assemblages.^67^ We quantified how extinction events restructure communities and if this increases the vulnerability of communities to future change. We used four traits (motility, tiering, feeding and body size), chosen for their relevance to species modes of life in the Bambach ecospace model, where they represent the realized eco-space of species^60^ and ease of acquisition from fossil material, for 115 marine taxa pre- and post-extinction fossil occurrence data represents marine communities comprising of macroinvertebrates, fish, and trace fossils (i.e., burrows and surface traces).^67^ The study interval extends across the early Toarcian extinction event (ETEE; ∼183 Ma) and occurrence data were collected from the Cleveland Basin, Yorkshire, UK. We categorized traits and obtained 18 trait values (refer to Table S1). For both time periods, we performed Pearson’s correlation analysis and visualized the networks (Figure 3). We calculated null models to ensure the metrics were significantly different from random (S2: Appendix).

*Pre-Extinction Event* degree centrality was not different from a null distribution; however, *Post-Extinction Event* degree centrality was significantly higher than random (*p* < 0.05) (Table S2). The net modularity of both the *Pre-* and *Post-Extinction Event* networks was significantly lower than random (*p* < 0.05), indicating that the modules formed were likely to have ecological meaning. Edge density was highest pre-extinction (0.22) and dropped following the extinction event (0.16). These fluctuations in edge density, along with the loss of one trait during the extinction event (deposit feeding) and a shift in keystone traits (Figure 3), could indicate a potential reduction in resilience *Post-Extinction Event*.

We propose that trait networks can reveal the functional redundancy of a community and therefore indicate how resilient it is likely to be to future disturbance. For example, complex, highly diverse and functionally redundant communities such as tropical coral reef communities are more likely to have complex, highly connected networks than temperate reef communities (Box 1). Tropical reef communities tend to have high functional redundancy,^19^ as is evident by their lack of reliance on one or two “keystone” traits, unlike temperate communities (Box 1). Increases in functional vulnerability are often seen in communities following an extinction event,^76^ where all functional groups are still represented within the community, but with very few species in each group—essentially the community is working with a limited “skeleton crew”^15^^,^^64^^,^^67^ (Box 2). The inclusion of species abundance information, such as the inclusion of fish biomass (Box 1), likely increases the accuracy of trait networks as this will reduce the likelihood of the importance of rare traits being over-inflated.

As trait modules reveal traits that co-occur more often with each other than other traits in the network, modules may represent functional groups or life history strategies. In very functionally redundant communities, functional groups or modes of life are harder to decipher from trait networks as there are many species with overlapping niche spaces and many, often similar, modes of life. Life history strategies are often described as existing on a continuum from r-selected species with quick life cycles to K-selected species with slower life cycles.^77^^,^^78^ There are likely many strategies, modes of life, or “niches” that exist in-between these too extremes, especially in diverse and functionally redundant communities. As conditions become more marginal this is less true, and successful modes of life appear in response to the “disturbance” or marginality of the environment. These successful modes of life are often one of the two extremes: r-selected or K-selected. For example, the “skeleton” crew described above following the ETEE consists of two main modules (Box 2: Figure 3). One of these modules consists of large and gigantic predators (Box 2), i.e., K-selected species that likely had a slower life cycle with one or two offspring.^77^ The other module contains smaller, suspension feeders and grazers (Box 2), which are more likely to be broadcast spawners with a faster life cycle, i.e., r-selected.^77^ Similar to as in modern communities along an environmental gradient, such as in Box 1, communities at the more marginal end of the gradient split more clearly into r-K extremes. In our Box 1 example there are three main modules: one r-selected module containing traits such as short PLD, small maximum length and scatterers, one K-selected module with traits such as a long PLD, large maximum length, predators, brooders and live bearers, and one “in-between” module with medium PLD, medium length, demersal spawning and piscivore (Box 1). The “in-between” module perhaps indicates a generalist survival mode, as we know there is an increase in generalist reef fish in the temperate region.^35^ Therefore, we propose more vulnerable trait networks may be simpler but also their modules will more clearly indicate trade-offs in life history strategies.

## Prospects of trait networks

We propose that trait networks provide an exciting and novel framework for exploring the effects of climate change on trait relationships within marine communities. Through the modularity and connectedness metrics derived from trait networks,^40^^,^^43^ there is the potential that the resilience of marine communities to environmental change can be quantified, with high levels of modularity, edge density, and degree centrality indicating high resilience within a community. In turn, these metrics can be used to identify structural patterns related to robustness and resilience, and possibly allow for the prediction of how communities will respond to future changes, based on the structural patterns of modern communities. However, it is important to consider that the generality of direct relationships between trait correlations and resilience remains untested across multiple communities.

Metrics such as node degree will allow the identification of traits central to marine community structure,^40^^,^^43^ such as the centrality, and therefore importance of piscivores in temperate reef fish community structure (Box 1). Investigating the relationships between these traits and the species they belong to will also help explore community resilience. If keystone traits have low redundancy or are related to particularly vulnerable species, the community could be vulnerable to disturbances.^37^ Keystone traits will likely change with changes to community structure and changes to environmental conditions,^37^ but particularly consistent keystone traits could be thought of as resilient traits. It is important to consider the choice of traits selected for trait networks, as they could limit their applicability and will be highly dependent on the question being asked.^37^ Traits are often correlated and it is difficult to untangle direct relationships from indirect and casual trait-trait relationships.^37^

However, comparisons between species co-occurrence networks and trait networks may reveal the resilience of trait networks (e.g., when keystone species go extinct, what effect would this have on the underlying trait network?), as long as traits are carefully selected, and research questions considered. Identifying vulnerable or important traits and the species they relate to can be used to advise conservation strategies.

## Conclusions

Trait networks can provide insights into structural changes and variations in trait-trait relationships in response to environmental changes. Trait networks can be analyzed using modern data, such as the example in Box 1, where strong temperature gradients can act as a proxy to study climate change. Analyses of trait-trait relationships can also be achieved using fossil occurrence data, where traits are known (Box 2), to look at trait changes through time in response to environmental disturbances. With further analyses, trait network metrics have the potential to quantify the resilience of communities and their ability to recover from environmental disturbances and highlight vulnerable marine communities.

## Limitations of the study

Trait network complexity and metrics likely vary greatly depending on species and community ecology and dynamics. Here, we provide two examples of how trait networks can be utilized to explore trait relationships and the effect this may have on community resilience. We do not suggest that these metrics can definitively quantify resilience across all possible marine communities or ecosystems.

## Acknowledgments

This research was funded by the Leverhulme Trust Extinction Studies Doctoral Training Programme. Japanese reef fish data collection was funded by grants from a Japanese Society for the Promotion of Science (JSPS) “Zuno-Junkan” grant entitled “Studies on origin and maintenance of marine biodiversity and systematic conservation planning” to J.D.R., and an 10.13039/100007615Australian Research Council Centre of Excellence for Environmental Decisions grant (CE110001014) and an EU Marie Sklodowska Curie Fellowship (TRIM-DLV-747102) to M.B. The Toarcian Extinction Event data collection and analysis was funded by the Palaeontological Association Undergraduate Research Bursary (PA-UB01703) and a NERC/NSF Pushing the Frontiers grant (NE/X015025/1) to A.D.

## Author contributions

Conceptualization, C.G.C.; methodology, C.G.C.; formal analysis, C.G.C.; investigation—Box 1 data, J.D.R. and M.B.; investigation—Box 2 data, A.M.D.; data curation, C.G.C.; writing—original draft, C.G.C.; writing—review and editing, C.G.C., A.M.D., J.D.R., and M.B.; supervision, M.B. and A.M.D.

## Declaration of interests

The authors declare no conflicts of interest.
