# Supplementary material for: Trait networks: Assessing marine community resilience and extinction recovery
Source: iScience. 2024 Sep 16;27(10):110962. doi: 10.1016/j.isci.2024.110962 (PMC11490707; doi:10.1016/j.isci.2024.110962)
Supplement: Document S1. Tables S1 and S2 [file mmc1.pdf]

iScience, Volume *27*

Supplemental information

Trait networks: Assessing marine community resilience and extinction recovery

Charlotte G. Clay, Alexander M. Dunhill, James D. Reimer, and Maria Beger

Supplementary Information

Table S1 – The four shallow marine community trait categories, all possible trait values, and their abbreviations used to assess trait correlations during the Toarcian extinction event.

Trait Category	Trait Value
**Feeding**	Deposit
	Grazer
	Mining
	Predator
	Suspension
**Motility**	Facultative
	Fast
	Slow
	Non-mobile
**Size**	Gigantic
	Very Large
	Large
	Medium
	Small
	Tiny
**Tiering**	Epifaunal
	Infaunal
	Pelagic

Table S2 – Network metrics for Toarcian Extinction Event trait networks. Observed network metrics are shown, alongside the mean of the 100 random networks created using the

Erdos-Renyi model and the p-values that indicate if the observed network metrics were significantly different from the null distribution.

	Time	Degree Centrality	Edge Density	No of Modules	Net Modularity
Observed Network	Pre-Extinction	0.319539	0.219048	4	0.50189
Random Network Mean		0.956357	1.234478	0.923403	1.015526
p-value		0.19	0.54	0.68	0.01**
Observed Network	Post-Extinction	0.773398	0.161765	4	0.532025
Random Network Mean		0.727636	1.267315	1.267801	1.201775
p-value		0.04*	0.65	0.85	0.04*

S1: Appendix

Box 1: Methods

Fish biomass and trait information were obtained from Clay *et al.*, (2023). We used seven of the 31 sites surveyed in the Clay *et al.*, (2023) paper, those identified as Tropical (four sites) and Temperate (three sites) in the cluster analysis performed in Clay *et al.*, (2023), each site contains 3-5 transect replicates. Five traits for 183 reef fish species were identified. The traits chosen were maximum length, pelagic larval duration, trophic level, water column position, and reproductive mode.

A presence/ absence trait matrix was built for all 183 species identified. Community-weighted means (CWM) were calculated using the FD package (v.1.0.12.1) in R (Laliberté *et al.*, 2014) using the biomass of fish species by transect and the trait matrix. Pearson's correlation was used to create a trait-trait relationship from the CWM matrix. A threshold of $p < 0.05$ where correlations above this threshold were assigned as 0 and those below were assigned as 1, indicating the presence and absence of significant trait-trait relationships (He *et al.*, 2020). The resulting trait-trait matrix was visualised as a network of correlations in igraph (v.1.4.1) (Csardi, 2013). Following network construction, we calculated four network metrics using igraph (v.1.4.1): degree centrality, edge density, modularity and node degree.

We applied bootstrapping to the trait dataset and generated 1000 networks for each of the regions: Tropical and Temperate. We calculated network metrics for each of the 2000 networks and compared each metric between the regions using Mann-Whitney-U tests.

S2: Appendix

Box 2: Methods

Toarcian extinction event (ETEE; ~ 183 Ma) fossil occurrence data was obtained from Dunhill *et al.*, (2022). We used four traits (motility, tiering, feeding and body size), chosen for their relevance to species modes of life in the Bambach ecospace model, where they represent the realised eco-space of species (Bambach, Bush and Erwin, 2007) and ease of acquisition from fossil material, for 115 marine taxa pre- and post-extinction. Fossil occurrence data represents marine communities comprising of macroinvertebrates, fish, and trace fossils (i.e. burrows and surface traces) (Dunhill *et al.*, 2022). We categorised traits and obtained 18 trait values (refer to Table S1).

A presence/ absence trait matrix was built for all 115 taxa identified. A presence/absence site by-species matrix was constructed and combined with the trait matrix to create a trait-trait matrix. Pearson's correlation was used to create a trait-trait relationship matrix from the trait-trait matrix. A threshold of $p < 0.05$ where correlations above this threshold were assigned as 0 and those below were assigned as 1, indicating the presence and absence of significant trait-trait relationships (He *et al.*, 2020). The resulting trait-trait matrix was visualised as a network of correlations in igraph (v.1.4.1) (Csardi, 2013). Following network construction, we calculated four network metrics using igraph (v.1.4.1): degree centrality, edge density, modularity and node degree.

To determine if the trait co-occurrences significantly differ from random, random networks were created and compared to the observed network (Lau *et al.*, 2017). We used the Erdos-Renyi model (Renyi, 1959) in igraph (v.1.4.1) to generate 100 random networks for each period (*Pre-* and *Post-Extinction event*) with the same number of nodes and edges as the observed network but with a randomised structure (Baiser, Elhesha and Kahveci, 2016). The observed network metrics were then compared to the null distribution of the network metrics using a permutation test (Fredrickson and Chen, 2019).

References

Baiser, B., Elhesha, R. and Kahveci, T. (2016) ‘Motifs in the assembly of food web networks’, *Oikos*, 125(4), pp. 480–491. Available at: https://doi.org/10.1111/oik.02532.

Bambach, R.K., Bush, A.M. and Erwin, D.H. (2007) ‘Autecology and the Filling of Ecospace: Key Metazoan Radiations’, *Palaeontology*, 50(1), pp. 1–22. Available at: https://doi.org/10.1111/j.1475-4983.2006.00611.x.

Clay, C.G. *et al.* (2023) ‘Variation in functional composition of reef fishes along a tropical-to-temperate gradient’, *Journal of Biogeography*, n/a(n/a). Available at: https://doi.org/10.1111/jbi.14768.

Csardi, M.G. (2013) ‘Package “igraph”’, *Last accessed*, 3(09), p. 2013.

Dunhill, A.M. *et al.* (2022) ‘Extinction cascades, community collapse, and recovery across a Mesozoic hyperthermal event’. bioRxiv, p. 2022.06.13.495894. Available at: https://doi.org/10.1101/2022.06.13.495894.

Fredrickson, M.M. and Chen, Y. (2019) ‘Permutation and randomization tests for network analysis’, *Social Networks*, 59, pp. 171–183. Available at: https://doi.org/10.1016/j.socnet.2019.08.001.

He, N. *et al.* (2020) ‘Plant Trait Networks: Improved Resolution of the Dimensionality of Adaptation’, *Trends in Ecology & Evolution*, 35(10), pp. 908–918. Available at: https://doi.org/10.1016/j.tree.2020.06.003.

Laliberté, E. *et al.* (2014) ‘Package “FD”’, *Measuring functional diversity from multiple traits, and other tools for functional ecology* [Preprint].

Lau, M.K. *et al.* (2017) ‘Ecological network metrics: opportunities for synthesis’, *Ecosphere*, 8(8), p. e01900. Available at: https://doi.org/10.1002/ecs2.1900.

Renyi, E. (1959) ‘On random graph’, *Publicationes Mathematicate*, 6, pp. 290–297.
