# Supplementary material for: Trait networks: Assessing marine community resilience and extinction recovery
Source: iScience. 2024 Sep 16;27(10):110962. doi: 10.1016/j.isci.2024.110962 (PMC11490707; doi:10.1016/j.isci.2024.110962)
Supplement: Data S1. Methods [file mmc2.pdf]

Trait Networks: Assessing Marine Community Resilience and Extinction Recovery

Charlotte G. Clay[1], Alexander M. Dunhill[2], James D. Reimer[3,4], Maria Beger[1,5]

STAR Methods

KEY RESOURCES TABLE

REAGENT or RESOURCE	SOURCE	IDENTIFIER
Deposited Data		
Box 1 Data: Japanese reef fish community weighted mean data	https://doi.org/10.5281/zenodo.13691990<br>https://datadryad.org/stash/dataset/doi:10.5061/dryad.ns1rn8q03	
Box 2 Data: TEE Fossil community data and trait data	https://doi.org/10.5281/zenodo.13691990	

Method Details

Fish biomass and trait information were obtained from Clay *et al.,* (2024)[1]. We used seven of the 31 sites surveyed in the Clay *et al.,* (2024)[1] paper, those identified as Tropical (four sites) and Temperate (three sites) in the cluster analysis performed in Clay *et al.,* (2024)[1], each site contains 3-5 transect replicates. Five traits for 183 reef fish species were identified. The traits chosen were maximum length, pelagic larval duration, trophic level, water column position, and reproductive mode.

Quantification and Statistical Analysis

A presence/ absence trait matrix was built for all 183 species identified. Community-weighted means (CWM) were calculated using the FD package (v.1.0.12.1) in R [2], using the biomass of fish species by transect and the trait matrix. Pearson's correlation was used to create a trait-trait relationship from the CWM matrix. A threshold of $p < 0.05$ where correlations above this threshold were assigned as 0 and those below were assigned as 1, indicating the presence and absence of significant trait-trait relationships[3]. The resulting trait-

trait matrix was visualised as a network of correlations in igraph (v.1.4.1)[4]. Following network construction, we calculated four network metrics using igraph (v.1.4.1): degree centrality, edge density, modularity and node degree.

We applied bootstrapping to the trait dataset and generated 1000 networks for each of the regions: Tropical and Temperate. We calculated network metrics for each of the 2000 networks and compared each metric between the regions using Mann-Whitney-U tests.

Box 2

Method Details

Toarcian extinction event (ETEE; ~ 183 Ma) fossil occurrence data was obtained from Dunhill *et al.,* (2022, Pre-print) [5]. We used four traits (motility, tiering, feeding and body size), chosen for their relevance to species modes of life in the Bambach ecospace model, where they represent the realised eco-space of species[6] and ease of acquisition from fossil material, for 115 marine taxa pre- and post-extinction. Fossil occurrence data represents marine communities comprising of macroinvertebrates, fish, and trace fossils (i.e. burrows and surface traces)[5]. We categorised traits and obtained 18 trait values (refer to Table S1).

Quantification and Statistical Analysis

A presence/ absence trait matrix was built for all 115 taxa identified. A presence/absence site by-species matrix was constructed and combined with the trait matrix to create a trait-trait matrix. Pearson's correlation was used to create a trait-trait relationship matrix from the trait-trait matrix. A threshold of $p < 0.05$ where correlations above this threshold were assigned as 0 and those below were assigned as 1, indicating the presence and absence of significant trait-trait relationships[3]. The resulting trait-trait matrix was visualised as a network of correlations in igraph (v.1.4.1)[4]. Following network construction, we calculated four

network metrics using igraph (v.1.4.1): degree centrality, edge density, modularity and node degree.

To determine if the trait co-occurrences significantly differ from random, random networks were created and compared to the observed network[7]. We used the Erdos-Renyi model[8] in igraph (v.1.4.1) to generate 100 random networks for each period (*Pre-* and *Post-Extinction event*) with the same number of nodes and edges as the observed network but with a randomised structure[9]. The observed network metrics were then compared to the null distribution of the network metrics using a permutation test[10].

References

1. Clay, C.G., Reimer, J.D., Sommer, B., Cook, K.M., Mizuyama, M., Obuchi, M., Kawamura, I., Kise, H., and Beger, M. (2024). Variation in functional composition of reef fishes along a tropical-to-temperate gradient. J. Biogeogr. *51*, 454–466. https://doi.org/10.1111/jbi.14768.

2. Laliberté, E., Legendre, P., Shipley, B., and Laliberté, M.E. (2014). Package 'FD.' Meas. Funct. Divers. Mult. Traits Tools Funct. Ecol.

3. He, N., Li, Y., Liu, C., Xu, L., Li, M., Zhang, J., He, J., Tang, Z., Han, X., Ye, Q., et al. (2020). Plant Trait Networks: Improved Resolution of the Dimensionality of Adaptation. Trends Ecol. Evol. *35*, 908–918. https://doi.org/10.1016/j.tree.2020.06.003.

4. Csardi, M.G. (2013). Package 'igraph.' Last Accessed *3*, 2013.

5. Dunhill, A.M., Zarzyczny, K., Shaw, J.O., Atkinson, J.W., Little, C.T.S., and Beckerman, A.P. (2022). Extinction cascades, community collapse, and recovery across a Mesozoic hyperthermal event. Preprint at bioRxiv, https://doi.org/10.1101/2022.06.13.495894 https://doi.org/10.1101/2022.06.13.495894.

6. Bambach, R.K., Bush, A.M., and Erwin, D.H. (2007). Autecology and the Filling of Ecospace: Key Metazoan Radiations. Palaeontology *50*, 1–22. https://doi.org/10.1111/j.1475-4983.2006.00611.x.

7. Lau, M.K., Borrett, S.R., Baiser, B., Gotelli, N.J., and Ellison, A.M. (2017). Ecological network metrics: opportunities for synthesis. Ecosphere *8*, e01900. https://doi.org/10.1002/ecs2.1900.

8. Erdös, P., and Rényi, A. (1959). On Random Graphs I. Publ. Math. *6*, 290–297.

9. Baiser, B., Elhesha, R., and Kahveci, T. (2016). Motifs in the assembly of food web networks. Oikos *125*, 480–491. https://doi.org/10.1111/oik.02532.

10. Fredrickson, M.M., and Chen, Y. (2019). Permutation and randomization tests for network analysis. Soc. Netw. *59*, 171–183. https://doi.org/10.1016/j.socnet.2019.08.001.

80
